# Exercise Training Favorably Modulates Gene and Protein Expression That Regulate Arterial Cholesterol Content in CETP Transgenic Mice

**DOI:** 10.3389/fphys.2018.00502

**Published:** 2018-05-08

**Authors:** Paula R. Pinto, Karolline S. da Silva, Rodrigo T. Iborra, Ligia S. Okuda, Diego Gomes-Kjerulf, Guilherme S. Ferreira, Adriana Machado-Lima, Debora D. F. M. Rocco, Edna R. Nakandakare, Ubiratan F. Machado, Maria L. Correa-Giannella, Sergio Catanozi, Marisa Passarelli

**Affiliations:** ^1^Laboratório de Lípides LIM 10, Hospital das Clínicas da Faculdade de Medicina da Universidade de São Paulo, São Paulo, Brazil; ^2^Faculdade de Ciências Biológicas e da Saúde (FCBS), Universidade São Judas Tadeu, São Paulo, Brazil; ^3^Mestrado em Ciências do Envelhecimento, Universidade São Judas Tadeu, São Paulo, Brazil; ^4^Laboratório de Fisiologia do Exercício Físico e Saúde da Faculdade de Educação Física e Esportes da Universidade Santa Cecília, São Paulo, Brazil; ^5^Departamento de Fisiologia e Biofísica, Instituto de Ciências Biomédicas, Universidade de Sao Paulo, São Paulo, Brazil; ^6^Laboratório de Carboidratos e Radioimunoensaio LIM 18, Hospital das Clínicas da Faculdade de Medicina da Universidade de São Paulo, São Paulo, Brazil; ^7^Programa de Pós-Graduação em Medicina, Universidade Nove de Julho, São Paulo, Brazil

**Keywords:** exercise training, macrophage cholesterol efflux, reverse cholesterol transport, cholesterol ester transfer protein, atherosclerosis

## Abstract

Aerobic exercise training (AET) improves the reverse cholesterol transport (RCT) in cholesteryl ester transfer protein-transgenic (CETP-tg) mice. We aimed at investigating the role of AET in the expression of genes and proteins involved in lipid flux in the aorta and macrophages of CETP-tg mice. Three-month-old male mice were randomly divided into trained (T; treadmill 15 m/min; 30 min/day) and sedentary (S) groups. After 6 weeks, peritoneal macrophages and the aortic arch were obtained immediately (0 h) or 48 h after the last exercise session. mRNA was determined by RT-qPCR, protein levels by immunoblot and ^14^C-cholesterol efflux determined in macrophages. AET did not change body weight, plasma cholesterol, triglycerides, glucose and CETP activity. In macrophages, at time 0 h, a higher expression of genes that encode PPAR gamma, ABCA-1 and a lower expression of MCP-1 and IL-10, was observed in T as compared to S. After 48 h, lower expressions of MCP-1 and PPAR gamma genes were observed in T mice. Increase in ABCA-1, SR-BI and IL-6 and decrease of LOX-1, MCP-1, TNF and IL-10 gene expression was observed in the aorta of T compared to S mice (0 h) and LOX-1 and MCP-1 remained diminished after 48 h. The protein level of MCP-1 and SR-BI in the aortic arch was unchanged in T animals after 48 h as compared to S, but LOX-1 was reduced confirming data of gene expression. The apo A-I and the HDL_2_ mediated-cholesterol efflux (8 and 24 h) were not different between T and S animals. In the presence of CETP, AET positively influences gene expression in the arterial wall and macrophages of CETP-tg mice contributing to the RCT and prevention of atherosclerosis. These changes were perceptible immediately after the exercise session and were influenced by the presence of CETP although independent of changes in its activity. Reductions in gene and protein expression of LOX-1 were parallel and reflect the ability of exercise training in reducing the uptake of modified LDL by the arterial wall macrophages.

## Introduction

Regular physical exercise reduces all-cause mortality and notably those related to cardiovascular outcomes (Lee et al., 2014; Schnohr et al., 2015). The beneficial effects are related to the prevention and or amelioration of cardiovascular risk factors such as dyslipidemia, hypertension, diabetes mellitus, and endothelial dysfunction (Ficker et al., 2010; Cornelissen and Smart, 2013; Umpierre et al., 2013; Guizoni et al., 2016). Exercise reduces plasma triglycerides (TG), small dense low-density lipoprotein (LDL), increases high-density lipoprotein cholesterol (HDLc), and apolipoprotein A-I (Halverstadt et al., 2007; Imamura et al., 2013; Sondegaard et al., 2014). In addition, it improves reverse cholesterol transport (RCT), a system that prevents atherogenesis by clearing the excess cholesterol from the arterial wall allowing its secretion into bile and feces excretion (Nijstad et al., 2011; Rocco et al., 2011; Pinto et al., 2015). Lipid-poor apo A-I and nascent pre-beta HDL interact with the ATP binding cassete subfamily A member 1 (ABCA-1) in arterial wall macrophages removing free cholesterol that is esterified by the lecithin cholesterol acyltransferase (LCAT) (Calabresi and Franceschini, 2010). Larger HDL particles that accommodate esterified cholesterol (EC) interact with the ATP binding cassete transporter G-1 (ABCG-1) removing more cell cholesterol. In animals lacking the cholesteryl ester transfer protein (CETP), the scavenger receptor class B type I (SR-BI) in the liver can directly remove EC from HDL (Azhar and Reaven, 2002). On the other hand, humans and other animal species that express CETP also have the EC transferred to apoB-containing lipoproteins by an exchange with TG (Morton and Izem, 2014). In this case, apoB-lipoproteins are removed by the B-E receptors in the liver (Go and Mani, 2012). Although several clinical trials addressed the impact of CETP inhibition in the enhancement of plasma HDLc and cardiovascular end points results are unsatisfactory for the most it is still not clear how CETP inhibition can affect RCT (Barter et al., 2007; Schwartz et al., 2012; Reveal Collaborative Group et al., 2017; Lincoff et al., 2017).

Inflammation that accompanies and aggravates the atherosclerotic lesion development is favorably modulated by exercise in many aspects. Particularly, by reducing intracellular cholesterol accumulation and improving antioxidant defenses in the arterial wall, exercise reduces inflammatory pathways associated with oxidative stress in macrophages (Meilhac et al., 2001; Wu et al., 2014). In addition, in many but not all cases, exercise increases HDL that is known for its antioxidant and anti-inflammatory actions (Kraus et al., 2002; Halverstadt et al., 2007; Iborra et al., 2008).

Aerobic exercise training (AET) improves the *in vivo* RCT in CETP-tg mice demonstrated by the increased recovery of ^3^H- cholesterol from intraperitoneally injected-macrophages in the plasma, liver and feces. This was ascribed to a higher expression of the B-E receptor in the liver and to the enhanced plasma HDLc levels due to a higher expression of hepatic ABCA-1 that contributes to the generation of new HDL particles (Rocco et al., 2011). Nonetheless, it is still unclear the role of exercise in the expression of genes that control lipid flux and homeostasis in macrophages of CETP-tg mice. Hence, we analyzed the role of an AET protocol in the modulation of genes and proteins that control inflammation, oxidative stress, lipid flux in the aorta and peritoneal macrophages and vasodilation in aorta of CETP-tg mice that may contribute to the exercise benefit on the RCT. In addition, we measured the apo A-I and HDL- mediated cholesterol efflux from peritoneal macrophages.

## Materials and Methods

### Animals and Treadmill Training Protocol

Inbred C57BL/6J transgenic mice homozygous for human CETP created by Dr. AR Tall’s laboratory were generously provided by Dr. HCF Oliveira (University of Campinas, São Paulo, Brazil) and were housed in a conventional animal facility at 22 ± 2°C with 12 h light/12 h dark cycle. After weaning and during the experimental protocol, mice had free access to water and were fed regular chow *ad libitum* (Nuvilab-Nuvital, São Paulo, Brazil). Three-month-old CETP-tg male mice were submitted to run adaptation, described previously (Pinto et al., 2015), and then divided into trained (T; *n* = 52) and control group that was kept sedentary (S; *n* = 50). The T-group performed monitored aerobic exercise on treadmill at 15 m/min, 30-min per day, five times a week for 6 weeks. The animal care was performed in accordance to the National Research Council (US) Committee for the Update of the Guide for the Care and Use of Laboratory Animals (2011) and approved by Animal Care and Research Advisory Committee (Hospital das Clinicas of the University of São Paulo Medical School - CAPPesq #441/11).

### Plasma Analyses

Plasma total cholesterol (TC), TG and HDL-c were determined before and after the experimental protocol by enzymatic colorimetric kit (Labtest, Brazil) and glucose by Accu-Chek^®^ Performa glucometer (Roche, Brazil). CETP activity was determined by the transfer of ^14^C-cholesteryl oleate from human HDL to human VLDL and LDL, after incubation with plasma from CETP-tg mice (Escolà-Gil et al., 2001).

### Blood Pressure Measurement

Blood pressure (BP) was determined using RTBP 2045 model coupled to an RTBP 001 data acquisition, a Data Acquisition System Laboratory – DASYLab 7.0, (DASYTEC, National Instruments Company, NH, United States) and an analysis system (Kent Scientific Corporation, CT, United States).

### Lipoproteins Isolation and LDL Acetylation

Low-density lipoprotein (*d* = 1.019 to 1.063 g/mL) and HDL_2_ (HDL_2,_ *d* = 1.063 to 1.125 g/mL) were isolated by ultracentrifugation of plasma from human health donors. Protein content in such lipoproteins was determined by the Lowry method (Lowry et al., 1951). LDL was acetylated according to Basu et al. (1976). Human procedures were performed in accordance to the Declaration of Helsink and all volunteers signed an informed written consent form approved by The Ethical Committee for Human Research Protocols of Hospital das Clinicas, University of São Paulo Medical School (CAPPesq #441/11).

### Macrophage Cholesterol Efflux

The cholesterol efflux assay was performed as previously described (Pinto et al., 2015), utilizing peritoneal macrophages isolated from T and S animals. Cells were previously overloaded with acetylated LDL (50 μg/mL) and 0.3 μCi/mL ^14^C-cholesterol and then incubated with apo A-I (30 μg/mL; 8 h) or HDL_2_ (50 μg/mL; 24 h) in order to access the cholesterol efflux pathways mediated by, respectively, ABCA-1 and ABCG-1.

### Gene Expression

Immediately (0 h) or 48 h after the last session of exercise protocol, mice were euthanized and macrophages were removed from the peritoneal cavity and aortic arch was harvested. Tissues were macerated in liquid nitrogen and homogenized as proposed by RNeasy^®^ Mini Kit (Qiagen, Hilden, Germany). The expression of genes was determined by real time quantitative reverse transcription polymerase chain reaction (RT-qPCR) as described before by Pinto et al. (2015) according to Livak and Schmittgen (2001). Using primer by Applied Biosystems (Foster City, CA, United States), the following genes were analyzed: *Abca1* (Mm00442646_m1), *Abcg1* (Mm00437390_m1), *Nr1h3* (Mm01329744_g1), *Nr1h2* (Mm00437265_g1), *Pparg* (Mm01184322_m1), *Scarb1* (Mm00450234_m1), *Olr1* (Mm00454586_m1), *Cd36* (Mm01135198_m1), *Tnf* (Mm00450234_m1), *Il6* (Mm00450234_m1), *Il10* (Mm00450234_m1), *Ccl2* (Mm00441242_m1), *Nos3* (Mm004435217_m1) and *Cat* (Mm00443258_m1). A gene stability assay was performed and according to a ranking order, *Actb* (Mm00607939_s1) and *Gapdh* (Mm99999915_g1) were utilized as endogenous control, respectively for macrophages and aortic arch samples.

### Immunoblot

Protein levels of LOX-1, MCP-1, SR-BI, and ABCA-1 whose genes were affected after exercise were analyzed by immunoblot. Artery samples were homogenized with lysis buffer containing 50 mM Tris, 0.15 M NaCl, 1% Triton X-100, 1% sodium deoxycholate, 10 mM EDTA and 0.1% SDS plus protease inhibitors cocktail (Sigma P8340) in a tissue disruptor. Protein content was determined by the BCA (Pierce Biotechnology, Rockford, IL, United States) method. Due to limitation in protein amount from the animal’s aortic arch, analyses were carried out with 40 μg of total tissue protein utilizing only artery samples from animals killed after 48 h of the last exercise session. Electrophoresis in 12% T sodium dodecyl sulfate (SDS) polyacrylamide gel was carried out and proteins were transferred to PVDF membranes, followed by blocking unoccupied sites by incubation with PBS containing 5% skim milk and 0.05% Tween. Membranes were exposed to anti-LOX-1 (1:100; Sc11655, Santa Cruz Biotechnology, Santa Cruz, CA, USA); anti-MCP-1 (1:1000; 2029, Cell Signaling, Danvers, MA, USA), anti-SR-BI (1:1000; NB400-101, Novus Biologicals, Littleton, Co USA) and anti-ABCA-1 (1:50; kindly provided by Prof. Shinji Yokoyama from Chubu University, Kasugai, Japan) primary antibodies. Membranes were washed with PBS + 0.05% Tween and then incubated with horseradish peroxidase-linked secondary antibody (1:5000, 1:2000; 1:1000 and 1:1000, respectively – Life Technologies, Carlsbad, CA, United States). After reaction with ECL (WESTAR; Cyanagen, Bologna, Italy), the bands were captured by the ImageQuant 350 (GE Healthcare, Piscataway, NJ, United States) and the densities of the respective lanes, stained by Ponceau, were used for normalization. The results were expressed as arbitrary units, related to mean of the sedentary animal, which was set as 100.

### Statistical Analysis

Comparisons between S and T groups were done by the unpaired Student’s *t*-test. Graph Pad Prism version 5.0 (San Diego, CA, United States) was utilized and a *p*-value < 0.05, considered statistically significant.

## Results

**FIGURE 1:**
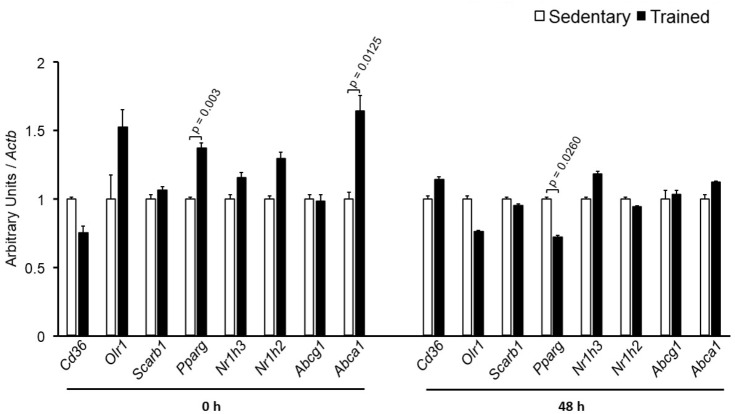
Expression of genes related to lipid flux in peritoneal macrophages harvested from sedentary (S) and trained (T) mice. Peritoneal macrophages from CETP-tg S and T were harvested immediately (0 h; S *n* = 6; T *n* = 6) or 48 h (S *n* = 7; T *n* = 7) after the last session of exercise. The expression of genes was analyzed by RT-qPCR and expressed as arbitrary units corrected per *Actb*. Comparisons were done by the unpaired Student *t*-test (mean ± SEM).

**FIGURE 2:**
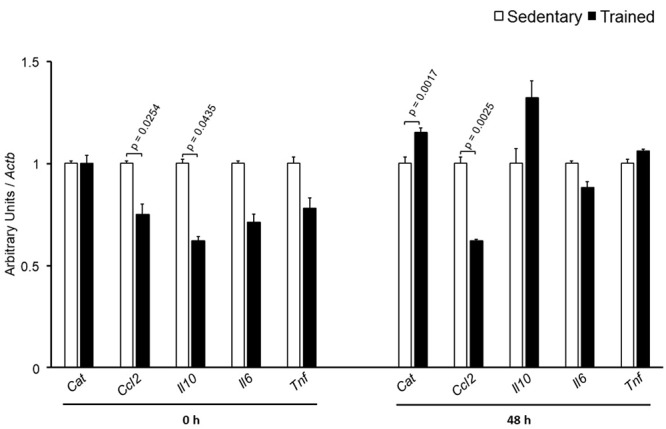
Expression of inflammation-related genes in peritoneal macrophages harvested from sedentary (S) and trained (T) mice. Peritoneal macrophages from CETP-tg S and T were harvested immediately (0 h; S *n* = 5; T *n* = 5) or 48 h (S *n* = 7; T *n* = 7) after the last session of exercise. The expression of genes was analyzed by RT-qPCR and expressed as arbitrary units corrected per *Actb*. Comparisons were done by the unpaired Student *t*-test (mean ± SEM).

The specific number of animals utilized for each experimental approach is detailed in the legends of figures. Body weight, plasma lipid and glucose levels, BP and CETP activity were similar between S and T animals in the basal and final periods (**Table 1**). As compared to S group, in peritoneal macrophages harvested from T mice immediately after the last exercise session (0 h) there was an increase in mRNA levels of genes involved in lipid flux, namely *Pparg* and *Abca1*. Nonetheless, after 48 h *Pparg* gene expression was reduced in T animals. Other genes involved in lipid flux were unchanged including *Cd36*, *Orl1*, *Scarb1*, *Nr1h3*, *Nr1h2*, and *Abcg1* (**Figure 1**). Regarding inflammatory gene response, it was observed a reduction in the expression of *Ccl2* (0 h and 48 h) and *Il10* (0 h, only) in macrophages harvested from T mice. No changes were observed in *Il6* and *Tnf* expressions in both periods after training. On the other hand, the expression of the antioxidant gene *Cat* was increased at 48 h in exercised animals (**Figure 2**).

**Table 1:** Body weight, plasma lipids and glucose, blood pressure and CETP activity in sedentary (S) and trained (T) CETP-tg mice.

	S (*n* = 50)		T (*n* = 52)	*p*
Body weight (g)	23.5 ± 1.8	Basal	23.5 ± 2.0	0.903
	25.2 ± 2.1	Final	24.7 ± 2.5	0.890
TC (mg/dL)	81 ± 18	Basal	80 ± 15	0.841
	75 ± 12	Final	76 ± 12	0.652
TG (mg/dL)	53 ± 14	Basal	47 ± 17	0.223
	48 ± 12	Final	48 ± 13	0.923
HDLc (mg/dL)	51 ± 5	Basal	53 ± 7	0.525
	50 ± 5	Final	56 ± 7	0.147
Glucose (mg/dL)	88 ± 12	Basal	89 ± 13	0.974
	107 ± 12	Final	108 ± 18	0.383
%CETP activity	31.8 ± 4.1	Basal	32.3 ± 8.2	0.794
	33.3 ± 7.2	Final	34.0 ± 9.7	0.798
BP (mmHg)	77 ± 3.4	Basal	84 ± 2.4	0.144
	77 ± 2.8	Final	75 ± 4.2	0.707

*Data expressed as mean ± SD; TC = total cholesterol; TG = triglycerides; HDLc = HDL cholesterol; blood pressure = BP*.

**FIGURE 3:**
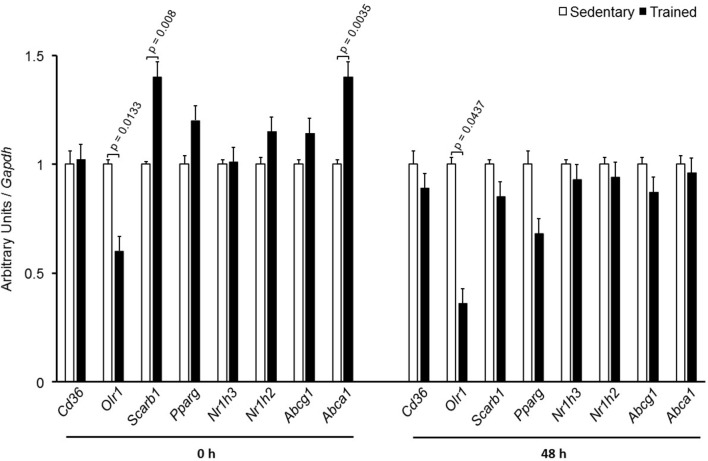
Expression of genes related to lipid flux in the aortic arch of sedentary (S) and trained (T) mice. The aortic arch was removed from CETP-tg S (*n* = 7) and T (*n* = 6) mice immediately (0 h) or 48 h after the last session of exercise. The expression of genes was analyzed by RT-qPCR and expressed as arbitrary units corrected per *Gapdh*. Comparisons were done by the unpaired Student *t*-test (mean ± SEM).

**FIGURE 4:**
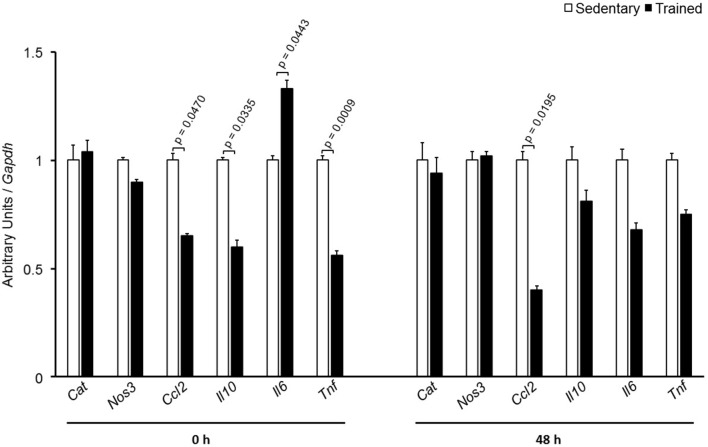
Expression of inflammation-related genes in the aortic arch of sedentary (S) and trained (T) mice. The aortic arch was removed from CETP-tg S (*n* = 7) and T (*n* = 6) mice immediately (0 h) or 48 h after the last session of exercise. The expression of genes was analyzed by RT-qPCR and expressed as arbitrary units corrected per *Gapdh*. Comparisons were done by the unpaired Student *t*-test (mean ± SEM).

**FIGURE 5:**
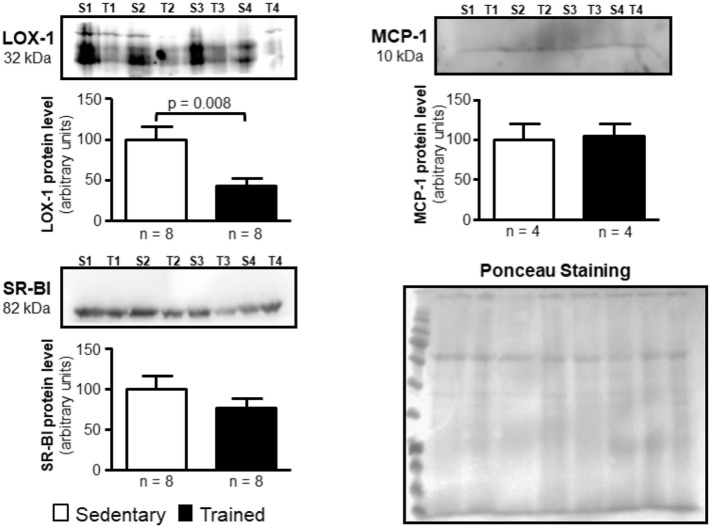
Expression of LOX-1, MCP-1, and SR-BI in the arterial wall of sedentary (S) and trained (T) animals. Immunoblot was performed by utilizing 40 μg of total protein from the aortic arch of S and T animals after 48 h of the last exercise session in order to confirm results obtained by RT-qPCR. Immunoblot was performed by using anti-LOX-1 (1:100); anti-MCP-1 (1:1000), anti-SR-BI (1:1000) and anti-ABCA-1 (1:50; not visualized) primary antibodies and horseradish peroxidase-linked secondary antibody (see section Materials and Methods for more details). The band densities of the respective lanes, stained by Ponceau, were used for normalization. The results were expressed as arbitrary units, related to mean of the sedentary animals, which was set as 100. Comparisons were done by the unpaired Student *t*-test (mean ± SEM). Representative images (*n* = 4–8, as indicated).

**FIGURE 6:**
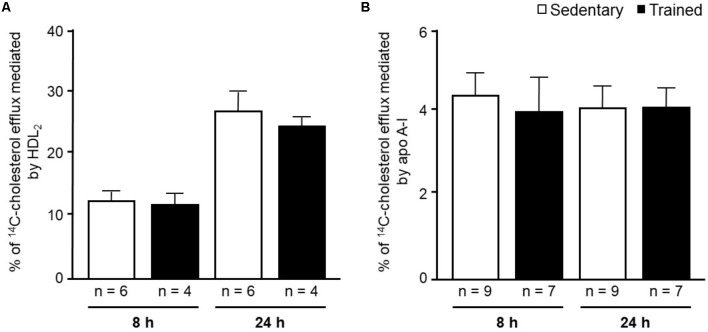
^14^C-cholesterol efflux from peritoneal macrophages harvested from sedentary (S) and trained (T) 48 h after the last exercise session. Macrophages were isolated from the peritoneal cavity of CETP-tg S (*n* = 9) and T (*n* = 7) mice 48 h after the last session of exercise. Cells were overloaded with acetylated LDL (50 μg/mL) and ^14^C-cholesterol (0.3 μCi/mL) and, after incubation with equilibrium media, were exposed to HDL_2_ **(A)** or apo A-I **(B)** for 8 h and 24 h. The % cholesterol efflux was determined as: ^14^C-cholesterol in media/^14^C-cholesterol in media + ^14^C-cholesterol remaining in cells x 100. Comparisons were done by the unpaired Student *t*-test (mean ± SD).

In the aortic arch, *Scarb1* and *Abca1* expressions were increased at time 0 h in aerobically T animals, but after 48 h they were no longer different. *Orl1* was reduced at both 0 h and 48 h. Other genes involved in lipid flux such as *Cd36, Pparg, Nr1h3*, *Nr1h2*, and *Abcg1* were similar between groups in both periods analyzed (**Figure 3**). Similarly to peritoneal macrophages, it was observed a reduced expression of *Ccl2* in the aortic arch of T animals both at 0 h and 48 h. *Tnf* and *Il10* were also reduced but only at 0 h. On the contrary, *Il6* gene expression increased in T animals (0 h, only) and *Nos3* and *Cat* were unchanged in both periods analyzed (**Figure 4**). Protein content was determined by immunoblot of total protein extracted from the artery of animals after 48 h of the last exercise session only, due to limitation of samples. As shown in **Figure 5**, the amount of LOX-1 was reduced in T animals as compared to S mice after 48 h, accordingly to the results observed in *Orl1* mRNA expression. No differences were observed regarding the amount of MCP-1 and SR-BI. We were unable to detect ABCA-1 protein levels by utilizing 40 μg of total protein from artery tissue. The cholesterol efflux was determined in peritoneal macrophages isolated from T and S animals that were incubated with apo A-I or HDL_2_-as lipid acceptors. The percentage of cholesterol removal was similar after 8 h and 24 h incubation with apo A-I and HDL_2_ (**Figure 6**).

## Discussion

To better explore how exercise improves RCT in CETP-tg mice we analyzed the effect of 6-week AET in the aortic arch and peritoneal macrophages expression of genes involved in lipid flux. CETP is expressed in humans and other animal species susceptible to atherosclerosis (Cheung et al., 1996; Morton and Izem, 2014). In this regard, mice with C57BL/6J background transgenic for human CETP have been widely used for studying atherogenesis.

Previous study from our group demonstrated that in WT mice the expression of genes involved in lipid uptake and efflux in the aortic arch and macrophages was not consistently changed by AET (Pinto et al., 2015). Those results are coherent with the thought that RCT is mostly influenced by systemic actions of exercise on HDLc plasma levels and receptors involved in the last stages of the RCT in the liver of WT mice.

In the present study, AET acutely enhanced the expression of genes involved in cholesterol efflux in peritoneal macrophages, *Abca-1* and *Pparg*. Pparg that was increased in time 0 h positively modulates *Abca1* gene transcription, reduces inflammation and ameliorates insulin sensitivity (Chawla et al., 2001; Bouhlel et al., 2007; Ogata et al., 2009). In mononuclear cells from healthy individuals peripheral blood a higher expression of *PPARG*, *NR1H3*, *ABCA1*, and *ABCG1* is described after a bout of cycling in 70% of VO_2máx_ or after 8 weeks of low-intensity exercise (Butcher et al., 2008; Yakeu et al., 2010). In another study exercise induced *CD36* that by increasing the uptake of oxidized lipids may activate PPARγ and downstream target genes such as ABC transporters and LXR (Chinetti et al., 2001; Thomas et al., 2012). Although in the present investigation the expression of *Abca-1* was no longer increased and *Pparg* was even reduced after 48 h of the last exercise session, we may consider the beneficial effects of regular exercise in preventing macrophage lipid accumulation. It was previously demonstrated that after a single bout of exercise the generation of nascent HDL particles by skeletal muscle is increased (Sviridov et al., 2003). These particles are known for their efficiency in removing cell cholesterol by interacting with ABCA-1 (Mulya et al., 2007). In accordance, Brites et al. (2004) described in soccer players an enhanced cholesterol efflux mediated by serum in comparison to sedentary controls, which was positively correlated with the concentration of circulating pre-beta HDL.

The expression of inflammatory genes was evaluated considering their role in atherogenesis. The chemokine MCP-1 gene, *Ccl2*, is activated by the accumulation of modified LDL in the arterial intima favoring monocyte recruitment and phenotype differentiation (Shih et al., 1999). A lower expression of *Ccl2* in peritoneal macrophages isolated from trained mice was observed in both periods, 0 h and 48 h, and may contribute to a lesser monocyte infiltration and to an increment in macrophage polarization into an anti-inflammatory M2 phenotype, as previously described (Yakeu et al., 2012). *Il-10* mRNA was reduced, which may be related to the diminished inflammatory stress elicited by exercise training even though we were unable to find changes in *Tnf* and *Il6* genes expressions in T mice.

Right away the exercise session it was observed an enhanced expression of catalase (*Cat*) in T animal macrophages. In spite of increasing oxidative stress, exercise training is demonstrated as effective in inducing antioxidants expression in the arterial wall prevailing the benefit of the latter in the prevention of atherosclerosis (Meilhac et al., 2001). Again, the expression of this gene was no longer observed at time 48 h.

Murine peritoneal macrophage has been utilized as a useful tool to study RCT, although the macrophage plasticity and heterogeneity reflects its susceptibility to the microenvironment influence (Cassado Ados et al., 2015). In other words, regarding lipid flux, peritoneal macrophages may not adequately represent macrophages from the arterial wall compartment, that are under specific metabolic influences that modulate atherosclerosis development. Following differentiation, macrophages may have distinct functional phenotypes according to diverse stimuli. Then, we looked at the aortic arch of S and T animals. In agreement to peritoneal macrophages, it was observed an increased expression of *Abca1* that favors cholesterol homeostasis in the arterial wall. ABCA-1 contributes to the major amount of cholesterol exported from cholesterol-loaded macrophages and mutations in *Abca1* gene cause atherosclerosis (Lee et al., 2011; van Capelleveen et al., 2015). The importance of ABCA-1 receptor, as well as the ABCG-1, has been confirmed in recent studies, in which the silencing of those receptors exacerbated the accumulation of cholesterol and inflammatory response in smooth muscle cells extracted from the aorta of mice (Cao et al., 2017; Castiglioni et al., 2017). In addition, *Scarb1* that encodes for SR-BI was enhanced after exercise session in T animals. SR-BI is a selective target of exercise in the liver mediating the uptake of cholesteryl ester form HDL in last phase of the RCT (Rocco et al., 2011). Here, we found that *Scarb1* is up-regulated by exercise and may constitute an additional route for cholesterol elimination from macrophages after HDL tethering. Although the action of SR-BI on the removal of cellular cholesterol is less representative than that observed by ABCA-1 and ABCG-1 receptors, its expression seems to play other determinant roles in atherogenesis, since its increased expression protects against development of atherosclerosis in mice, while its deletion induces plaque rupture in hypercholesterolemic animals fed a high fat diet (Vaisman et al., 2015; Hermann et al., 2016). After 48 h, those changes in *Abca1* and *Scarb1* were no longer different between T and S mice, and protein levels of ABCA-1 was undetectable and SR-BI was unchanged between T and S mice. The expression of *Orl1* was significantly reduced after 0 h and 48 h of exercise session and in agreement, LOX-1 protein level was reduced in T animals as compared to S after 48 h of exercise. This may contribute to a lesser uptake of oxidized LDL by endothelial cells and macrophages, which ultimately prevents atherosclerosis as described by others (Mehta et al., 2007; Chen et al., 2016). Then, the selective gene and protein expression observed in the present investigation seems to prevent cholesterol accumulation in the arterial wall compartment of T mice. No changes were observed in the expression of *Abcg1, Nr1h2, Nr1h3, Pparg*, and *Cd36* in the aortic arch of T as compared to S mice. The expression of *Ccl2* was similarly influenced by AET in macrophages and aortic arch. In the arteria, *Ccl2* was reduced at both 0 h and 48 h after exercise session. The inflammatory gene that encodes for TNF, *Tnf*, was also reduced and the same was observed regarding *Il10*. On the other hand, *Il6* mRNA was acutely increased in the aortic arch of T mice.

The *in vitro* evaluation of peritoneal macrophages harvested from S and T animals revealed no changes in the cholesterol efflux rate mediated by apo A-I or HDL_2_. The *in vitro* experiments were conducted in order to estimate cell changes induced by exercise without interference of HDL and apo A-I concentration and physicochemical properties that are likely to influence cell cholesterol removal *in vivo*. Nonetheless, we should bear in mind that besides having increased *Abca1* and *Pparg* expression, that positively modulates cholesterol efflux, the *in vitro* cell system lacks other pivotal components of the RCT. They include LCAT and CETP actions, the expression level of liver receptors and enzymes as well as variations in HDL particle number and functionality. All together, they create a centripetal cholesterol flow that favors its elimination in the bile and modulate the cholesterol content in the arterial wall (Hellerstein and Turner, 2014).

In conclusion, AET positively influences the expression of genes involved in lipid flux in peritoneal macrophages and arterial wall. The changes are perceptible immediately after the exercise session in trained CETP-tg mice and are independent of changes in CETP activity, although influenced by the presence of this protein since they were not observed in previous study dealing with WT mice trained in a similar exercise protocol (Pinto et al., 2015). Reductions in gene and protein expression of LOX-1 were parallel and reflect the ability of training in reducing the uptake of modified LDL by the arterial wall macrophages.

The pivotal role of CETP in modulating gene expression in macrophages and in the arterial wall was not detailed in the present investigation and needs further clarification. Conceivably, CETP is involved in the exchange of neutral lipids between lipoproteins by a bridging model, contributing to HDL remodeling and RCT. Nonetheless, other roles are described for CETP and include the selective uptake of EC-HDL by cells, including adipocytes, independently of SR-BI, B-E, and LRP receptors and the EC efflux out of cell membranes leading to intracellular accumulation of free cholesterol (Stein et al., 1986; Vassiliou and McPherson, 2004). Circulating CETP influences in insulin secretion and sensitivity in a dose dependent manner (Cappel et al., 2013; Guo et al., 2016). Altogether, these actions can modify the intracellular content of sterols and ultimately change the expression of genes in arterial wall macrophages. Besides, CETP has anti-inflammatory properties that are not strictly related to its lipid transfer activity but may help to prevent atherogenesis and modulate gene expression (Oliveira and de Faria, 2011), especially when combined with AET.

The pharmacological inhibition of CETP did not achieve success although increasing plasma HDL cholesterol levels. This may relate to several physiological processes in which CETP is involved. Although the mechanistic aspects were not clearly evidenced in the present investigation, the demonstration that the arterial gene expression is differently modulated by the presence of CETP is a new and original aspect of this investigation. Our results reinforce the importance of regular exercise in the prevention and regression of atherosclerosis in an animal model that resembles human lipoprotein metabolism conferred by the presence of CETP.

## Author Contributions

PP performed mice exercise training, carried out all analyses with the help of the other authors, and participated in experimental design and the manuscript preparation. KdS and DG-K helped in animal care and RT-qPCR analysis. GF helped in animal care and training. LO helped in cell culture and immunoblot. AM-L helped in cell experimental design. RI performed macrophage isolation and analysis and immunoblot. SC supervised animal surgery and participated in experimental design. DR participated in experimental design. EN helped in statistical analyses. UM and MC-G assisted and gave support in cell biology analyses. MP was responsible for experimental design, coordination of research, and preparation of the manuscript. All authors read and approved the final manuscript.

## Conflict of Interest Statement

The authors declare that the research was conducted in the absence of any commercial or financial relationships that could be construed as a potential conflict of interest.
